# Towards a Public Architectural History: Collective-Use Facilities and Community Engagement in Portugal and Spain

**DOI:** 10.1515/iph-2025-0002

**Published:** 2025-11-13

**Authors:** Ricardo Costa Agarez, Ana Mehnert Pascoal, Ivonne Herrera-Pineda

**Affiliations:** DINÂMIA’CET-Iscte, Centre for Socioeconomic and Territorial Studies, Instituto Universitário de Lisboa (ISCTE-IUL), Av. das Forças Armadas, 1649-026, Lisbon, Portugal

**Keywords:** architecture, built environment, co-construction of knowledge, collective memory, ethnography

## Abstract

The sustained use and reuse of existing buildings is key in addressing social inequality and reinforcing sustainability and resilience in peripheral, disadvantaged communities of the so-called developed world. Collective-use facilities built since the 1940s, the outcome of individual and common efforts, carry decades of service to communities and are repositories of both material and experiential values. Knowing their history of production and use is essential in reassessing their relevance for current and future needs: to be effective, this knowledge must be appropriable and relatable, co-created, and widely shared. This article discusses how such premises are put to the test in *Arquitectura Aqui*, a research and dissemination initiative underway in communities in Portugal and Spain. Using different cases in both countries to examine specific goals and methodologies, challenges and results, we suggest that local engagement in co-researching and co-narrating the past and present of buildings and their role in collective life, in a participation and dissemination platform, might contribute to putting into practice a public architectural history of community buildings.

## 1 Introduction: The Framework for a Public Architectural History Experiment

Until very recently, architectural historians generally conducted research in mostly the same manner as their colleagues across the humanities, which is to say, they did so largely working alone in libraries, museums, archives, and special collections. The single factor differentiating our work (…) was our need for on-site investigation [yet] we will continue to perform solitary investigations with a range of primary and secondary sources.1Dianne Harris, “Architectural History’s Futures,” *Journal of the Society of Architectural Historians* 74, no. 2 (2015): 147–51, quote on 147.

Dianne Harris’s pertinent analysis of the “futures” of architectural history highlighted its specificity and persistent traits; timidly, it also acknowledged a recent interest in “newer models of collaborative research practice that are based in the ‘distributive networks of expertise’ that tend to characterize digital humanities projects” and include “librarians, computer scientists and programmers, designers, and specialists in data curation.” These “new communities of scholars,” she wrote, find “greater value on the multiauthored and multilayered artifacts that result from complexly collaborative research.”2Ibid., 148.

Harris’s view, however, left out non-expert contributions: it is not easy to venture beyond the confines of academia and accredited expertise to include the layperson’s knowledge, while avoiding the pitfalls of subjectivity and upholding scientific credibility. Yet it is the responsibility of a socially relevant architectural history, we posit, to open its creation process to citizen participation. To address the “challenges in our efforts to engage the public in a more sophisticated, sustained, and robust set of dialogues” that Harris identified, we must find ways to *make architectural history public* – or, in other words, to practice forms of *public architectural history*. We need to work towards what Harris called “an architectural history for everyone and, ultimately, increased levels of public understanding about the significance and value of the built environment as it structures everyday life.”3Ibid., 150–51.

This article discusses the bases, methods, practice, and preliminary results of *Arquitectura Aqui – Community, Proximity, Action: Collective-Use Facilities in Portugal and Spain 1939-1985*, a research, writing, and dissemination initiative that seeks to act on these premises. After outlining its theoretical and methodological framework, we proceed to describe our approach to citizen participation in two sites – Penamacor, a small community in Portugal, and Cáceres, a larger but peripheral community in Spain – and reflect on the potential and limitations of our public architectural history proposition.

Buildings are not static and evolve throughout time, being shaped by human interaction and use. *Arquitectura Aqui* moves beyond the limited scope of conventional architectural history – the planning, design and opening stages of structures – to acknowledge the entire lifespan of collective-use buildings.4*Arquitectura Aqui* (online at https://arquitecturaaqui.eu) is an output of the research projects *ReARQ.IB – Built Environment Knowledge for Resilient, Sustainable Communities: Understanding Everyday Modern Architecture and Urban Design in [sic] the Iberian Peninsula (1939–1985)*, funded by the European Research Council (Starting Grant GA949686) and hosted by Iscte – University Institute of Lisbon, Portugal (2021–2026); and *The Architecture of Need: Community Facilities in Portugal 1945–1985*, funded by Fundação para a Ciência e a Tecnologia (PTDC/ART-DAQ/6510/2020) and hosted by Évora University, Portugal (2021–2025). It explores ways in which architectural and urban history might support more sustainable and resilient communities, by collectively creating, with community members, a new kind of built environment history.

We know precious little about the buildings where we reside, work, study, convalesce, and enjoy ourselves. With better knowledge – collectively constructed by those who research the buildings’ history and architecture, and those who created and experience them – we enable better informed decisions on how to manage, transform, and support the continuing use of such structures, at a time when sustainability imperatives demand that maintaining and revalorizing existing buildings should be a priority over the newly built. Locally indispensable, these structures were the outcome of collective efforts across decades, launched and supported by public and private entities and individuals with technical support and funding, and must be better known to continue to serve all.

This research follows the parallel, shared history of Portugal and Spain between 1939 and 1985 – from dictatorship to democratic transition and European integration – through the buildings where daily life unfolds: structures devoted to welfare and medical care, general and social services, minimum-rent housing, security, education, culture, and leisure. A multidisciplinary team effort involving architects, art and architecture historians, and anthropologists integrates detailed information and critical thinking on such objects, key in potential management and transformation initiatives, while also advancing scientific and historical knowledge on architecture and urban design in the Iberian countries. Data drawn from the archives and the memories and experiences of stakeholders, creators and users, are recorded and combined with the aim of co-creating a new narrative.

*Arquitectura Aqui* argues that 1) architectural history is essential for sustainably managing collective-use facilities, and 2) a new public form of such history is required: relevant, participated, appropriable. Our work begins with identifying sample communities in Portugal and Spain as test grounds for these premises, transposable to other geographies despite their specificity. Selected communities share commonalities – peripherality, interiority, depopulation, historical scarcity, invisibility for canonical built-environment history – while being representative of a diverse range of geographical, demographical, socio-economic and cultural traits; they are served by a meaningful set of public-use buildings from 1939 to 1985. Primary-source research (in central repositories) provides a preliminary knowledge basis for subsequent groundwork: in fieldwork missions to selected communities, further historical enquiry in local archives, libraries, and collections, and exchange findings with invested parties; we visit buildings and engage with users; we organize memory elicitation and sharing sessions with community groups; finally, we integrate the historical record and the output of local engagement in citizen-friendly, open-ended vignettes narrating the production and use life of buildings. This forms the most visible face of an online platform designed to be legible and attractive to laypeople, while also offering relevant information to anyone seeking solid knowledge on collective-use facilities on the Iberian Peninsula.

Contributions of researchers and community members are thereby weaved together in an exercise of narrating historical and experiential accounts that seeks to maintain its multivocality while ensuring public relevance: we draw on oral history, public history, and ethnography tools to pursue an ethical research practice.

Oral history can advance architectural history; buildings can be better understood by listening to those who have used them. We must learn to record, as Janina Gosseye put it, “the spatial knowledge embedded in interventions in buildings made post-completion by its inhabitants and users, as well as in the stories that they would be able to tell about buildings (…) often regarded as beyond the bounds of the discipline.”5Janina Gosseye, “A Short History of Silence: The Epistemological Politics of Architectural Historiography,” in *Speaking of Buildings: Oral History in Architectural Research*, ed. Janina Gosseye, Naomi Stead and Deborah van der Plaat (New York: Princeton Architectural Press, 2019), 12–27, quote on 11. If including disparate voices has become vital to oral history since the 1960s, it has been a relatively recent development in architectural history, where such voices are seldom other than those of the designers involved.6Recent work on this front includes David Adams, “Shaped by Memory: Oral Histories of Post-War Modernist Architecture,” Working Paper Series no. 12 (Birmingham: Birmingham University, 2012); Jesse Adams Stein, “The Co-construction of Spatial Memory. Enriching Architectural Histories of ‘Ordinary’ Buildings,” *Fabrications: The Journal of the Society of Architectural Historians, Australia and New Zealand* 24, no. 2 (2014): 178–97; Gosseye, “A Short History of Silence,” 9–23; and Gaia Caramellino, “Living Together (The Multiple) ‘Stories’ of an Ordinary Housing Development in Post-WWII Turin”, in *Contested Legacies: Critical Perspectives on Postwar Modern Housing*, eds. Martino Tattara and Andrea Migotto (Leuven: Leuven University Press, 2023), 133–54.

The potential of intersecting public history with architectural history, in turn, has been little explored: the growing number of events engaging citizens with buildings rarely include in-depth historical analysis.7For example, the Open House Worldwide network initiatives. See https://www.openhouseworldwide.org/. Yet this combined approach might broaden the scope of architectural history to integrate social dynamics and stories that are significant to the communities, while recognizing people as active agents in the history of these facilities. Accepted canons can be thereby put into perspective and official history becomes nuanced.

More than a history of architecture, this might then become a history of the community and its interaction with the built environment, encouraging its members to maintain their bonds with structures long at their service. Traditional participatory approaches often rely on interviews, surveys, and workshops. However, the influence that participants exert over the research process is as important as the methods. Anu Soikkeli pointed out that “a survey is not an actual participatory design method but a means of obtaining research material […] a survey is often used as a preliminary study of an inclusive method.”8Anu Soikkeli et al., “Challenges of Participatory Design in Apartment Buildings’ Renovation Projects in Finland,” *Journal of Housing and the Built Environment* 38, no. 3 (2023): 1889–1905. Therefore, a diversity of methods is needed to foster engagement through sustained dialogue and feedback loops. Ethnography provides a baseline for participation through its capacity to recover local voices and meanings, enabling a careful calibration between local knowledge and academic interpretation; it is key in directing our attention towards the practices and discourses of everyday life as sources of significant knowledge. Although long-term fieldwork has traditionally been required in ethnography, limited resources and changing circumstances have led scholars to explore alternative approaches, such as short visits and remote and digital communication, which enable the co-production of knowledge.9Chika Watanabe and Gökçe Günel, “Patchwork Ethnography,” *American Ethnologist* 51, no. 1 (2023): 131–39. Crucially, participation is both procedural and epistemic: research orientation, its narrative structure and representational choices determine whether the perspectives of participants are genuinely integrated. Consequently, prioritizing informants’ viewpoints and framing them as narrative agents, rather than as mere data sources, becomes central to an ethical and more horizontal research practice that seems paramount in built-environment awareness initiatives. As Thomas Yarrow argues, historic conservation should acknowledge the multiple negotiations between different agents, illustrating the existence of “specific people with specific understandings of what is ‘real’ and important about the past.”10Thomas Yarrow, “How Conservation Matters: Ethnographic Explorations of Historic Building Renovation,” *Journal of Material Culture* 24, no. 1 (2019): 3–21, quote on 18. This shift highlights the recognition of diverse experiences, values, motivations and interests as crucial in the relationship between architecture and everyday practices.11Albena Yaneva, “The Method of Architectural Anthropology,” in *Architectural Anthropology. Exploring Lived Space*, eds. Marie Stender, Claus Bech-Danielson, and Aina Landsverk Hagen (London and New York: Routledge, 2002), 13–29.

Making research outputs available in an online platform that disseminates research to a virtually global audience that tends to be “off the radar,” much beyond the reach of traditional academic work, is another facet of our public architectural history exercise. Public history supports the creation, registration, management, and conservation of sources.12Thomas Cauvin, “Campo Nuevo, Prácticas Viejas: Promesas y Desafíos de la Historia Pública,” *HISPANIA NUEVA. Revista de Historia Contemporánea* 1 (2020): 7–51, quote on 20. The *Arquitectura Aqui* platform does this by systematizing and analyzing often obscure, inaccessible archival sources, together with ignored buildings and unrecorded testimonies. The platform also encourages anyone interested in contributing data to participate in the narrative, whether by sending written testimony, or by sharing elements such as photographs of past moments in the building’s life. It extends these communities’ visibility and fosters dissemination, dialogue and collaboration, in line with one worthy remit of public history: rendering local pasts globally visible.13Serge Noiret and Thomas Cauvin, “Internationalizing Public History,” in *The Oxford Handbook of Public History*, eds. James B. Gardner and Paula Hamilton (New York: Oxford University Press: 2017), 25–43.

We recognize the challenges of gatekeeping. Yet we also defend the need for a degree of scrutiny by the research team over what is shared on an open access platform stemming from a publicly funded research project. As Serge Noiret and James B. Gardner have argued, in an era of wide proliferation of information and possibilities for immediate online sharing, researchers should keep a filtering role and act as mediators.14Serge Noiret, “Past Continuous: Digital Public History through Social Media and Photography”, in *What Is Public History Globally? Working With the Past in the Present*, eds. Paul Ashton and Alex Trapeznik (London: Bloomsbury Academic, 2019), 265–78; James B. Gardner, “Trust, Risk and Historical Authority: Negotiating Public History in Digital and Analog Worlds,” in *Making Histories*, eds. Paul Ashton, Tanya Evans, and Paula Hamilton (Berlin and Boston: De Gruyter: 2020), 59–67. This, in turn, prompts us to move beyond the confines of academic writing and technical jargon to achieve more inclusive communication. Moreover, we disseminate results and encourage collaboration via other means of outreach: targeted presentations and collective memory elicitation sessions, local radio and television pieces, social media, and European Researchers’ Night events are used to discuss the project and engage different audiences.15E.g. the action “Arquitectura Aqui na Escola: Onde Andou na Primária?,” where we ask European Researchers’ Night attendees to share their experience at elementary school while constructing a world map of such facilities.

## 2 Community Engagement Methodology

In our three-stage work process – preliminary sample survey, groundwork with communities, and communication –, we consider the second stage to be key in building the foundations for a public architectural history: this will therefore be our focus here.

Our preliminary sample of building records is collected in the archives of funding public bodies (government agencies devoted to public facilities and urban planning, housing and social welfare) and philanthropies, official publications and reports listing finished buildings, as well as other coeval literature, maps, and repositories. This sample is then amended and completed with the use of local sources including archives of municipal and regional services, local press, and institutional websites. Such sources generally provide data about the commission and construction process (who built what, on the request of whom and to what purpose, on whose design, at what cost). However, they are lacking in information regarding the use life of building and their social relevance in everyday community life: who eventually used and appropriated them, what adjustments were required and made, what emotional bonds were created between community members and facilities, how they served local social life.

We start with limited knowledge about how buildings were produced (sometimes) and appropriated by people (always), so we adopt an ethnography-through-oral-history strategy to elicit local architectural and social knowledge. Fieldwork targets people whose professional, experiential, or historical ties to the buildings are salient: technical staff, amateur historians, and older residents. Participants are recruited through two routes: pre-identified contacts via local history websites, and on-site spontaneous encounters with users and neighbors. After acquiring consent, we conduct flexible semi-structured interviews based on a general questionnaire that is adapted to each specific situation, leaving room for the interviewee to share information without interruption from the interviewer.16Informed consent is obtained from all individuals included in this study. This questionnaire takes the building as a starting point and traces planning and construction choices, information about promoters, evolving uses, and the wider social, economic and managerial contexts. Questions address: 1) the material characteristics and condition of the building; 2) its history, either through the agents and entities involved or through its uses and transformations; and 3) the memories and experiences originating in the building, including associated values and emotions.

**Figure 1:**
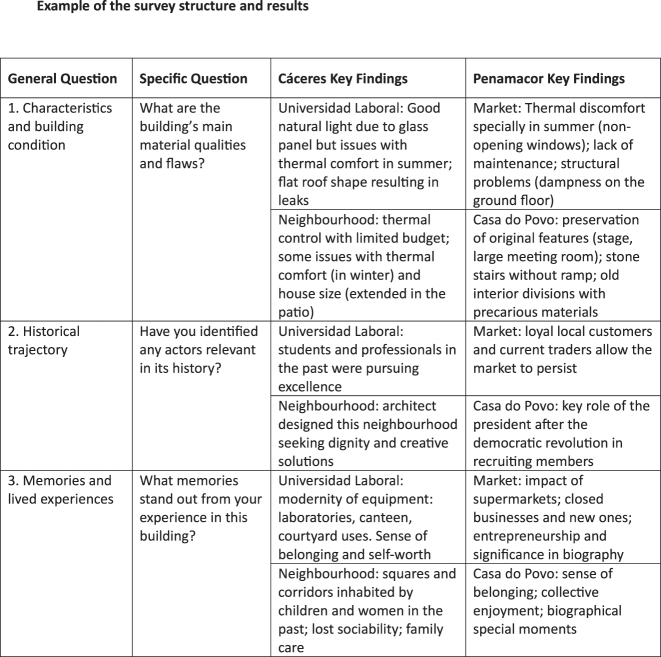
Survey structure and results.

The questionnaire (a summary of which is shown in Figure 1) is used primarily as a template for open and flexible questions that occasionally explore personal and affective memories. Copies of this questionnaire can be made available, personally or by email, to be filled later. The interviews are complemented by informal on-site conversations. Testimony collection is combined with observations of the buildings in use and users in action.

After talking to the people and recording the conversations, the most relevant information is compiled in written vignettes and made available to the public on the *Arquitectura Aqui* platform (when this has been agreed upon) in connection with the related documents, buildings, and communities. These discursive fragments can give an overview of the social life of the building and its transformations over time, while also providing relevant details about the construction process that might not have emerged in the archival research. Based on the information originating from these two essential sources – archival documentation and oral records – we proceed to weave together a succinct, layperson-friendly account of the life story of the building, from the first expression of need that prompted its creation to the present day, as the point of entry to each building’s webpage.

A final step, currently ongoing, involves communication with the public via the website, public events, media appearances, and press publications, as well as scientific articles for more specialized dissemination. In an online public event in March 2025, the full (albeit in-progress) version of the platform was presented to and discussed with stakeholders, local archives staff, and members of all the communities involved up to that point. Previous participants were among the over one-hundred attendees from Spain and Portugal and on this occasion, and afterwards, they were invited to explore this resource and pay particular attention to how the platform had incorporated their contributions. After the fieldwork stage we also re-contacted those informants who expressed special interest and genuine support in order to maintain a bond and clarify specific points. This keeps the process open to new outcomes while sustaining engagement and fostering ongoing collaboration, thus strengthening a collectively constructed narrative of architectural history. Details on devolution and collective construction of knowledge in the two case studies will be presented ahead.

## 3 Results: Cases in Penamacor (Portugal) and Cáceres (Spain)

We now focus on how this methodology was applied in specific circumstances, in two communities that we see as representative of our approach: Penamacor (Portugal) and Cáceres (Spain). These two fieldwork experiences reveal different social situations and prompt reflection on the potential reach of a public form of architectural history. We highlight paradigmatic and specific contributions made by individuals in these places that are relevant to the scope of the research from a public history perspective. It is important to note that full anonymity was preserved unless explicit consent to name disclosure was granted.

Penamacor and Cáceres are two of over 30 communities currently undergoing in-depth study in *Arquitectura Aqui*. Both were selected based on factors such as geographic location, dimension, distance from central governments, demographic changes since the 1940s, economic development, and the quantity and relevance of collective-use facilities built in the period. They are presented in this paper as examples of the diversity of methodological approaches and ways of engagement with communities. Each case covers two buildings that stood out of the 29 buildings studied in Penamacor and 52 in Cáceres.

The first of these communities, Penamacor (https://arquitecturaaqui.eu/pt/comunidades/21070/penamacor), is a small municipality in the administrative circumscription of Castelo Branco, in central Portugal, near the Spanish border. Geographically, Penamacor is marked by a contrast between mountainous and flat areas. Population decreases and migrations have been persistent since the 1950s. Currently, Penamacor is experiencing some population renewal through immigration from non-European countries (e.g. USA and Israel), although retaining younger generations is proving to be a challenge. The population is dispersed across the territory: only 4,745 inhabitants covered 546 km^2^ in 2022.17Data obtained at Instituto Nacional de Estatística (INE). Reliance on agriculture endures, as industry is scarce, and commerce is declining. In terms of collective-use facilities, several buildings were identified: local administration headquarters, fire station, post office, security forces outpost, primary schools, kindergarten, parish hall, market hall. In total, for the two buildings presented below – Casa do Povo and market hall –, fieldwork was carried out between September 2023 and May 2024 and included three semi-structured interviews, seven informal conversations, and one group discussion.

Engaging with the local community stemmed from initial contacts made during a field trip for archival research at the municipal archive.18Furthermore, archival research was conducted in central archives in Lisbon, namely the records of the former Directorate-General of National Buildings and Monuments, the historical archive of Land Planning and Urban Development, the Torre do Tombo national archives, and the archives of the Ministry of Labour, Solidarity and Social Security. In a casual conversation, the owner of the accommodation where one of the researchers was staying referred us to a personal contact. This contact became crucial as it proved to be perfectly suited to our intentions. We then met Francisco Abreu, who, from the beginning, enthusiastically collaborated with us and gradually became a key figure in the research process. Francisco is a writer and a retired secondary school philosophy teacher, as well as a Regional and Local History lecturer at Penamacor’s Academia Sénior, a learning and meeting space for senior citizens established through an informal grass-roots initiative. Francisco became our host in Penamacor and facilitated a large part of our fieldwork. He was indispensable in identifying local actors with a strong presence in collective memory and familiarity with the buildings, as well as mediating between different social spheres. In addition, Francisco shared clippings from local newspapers with vital information that we integrated onto the platform: it complemented historical data from the archives and conversations by identifying relevant actors and the (sometimes critical) stance of residents during construction processes.

Through Francisco, recognized as a trusted community actor, we were welcomed by the Junta de Freguesia, the smallest-scale local authority unit in the Portuguese political organization, the Casa do Povo community center, and the Municipal Museum, as our goals were in line with the proximity approach of these organizations. Similarly, the conversations we had with people, including our involvement with the senior academy, were only possible because of his active collaboration. This support can be attributed to Francisco’s personal background and his interest in local heritage and history, other than his genuine interest in our initiative. Indeed, he is actively involved in cultural activities in Penamacor and has held local political positions.

Crucially, we connected our interests with each other and, through him, with those of other people, creating a synergy between different people who valued the same issues from different perspectives. For instance, we co-organized a participation event during one of the local history sessions Francisco convened at the senior academy, with ca. 30 participants with different backgrounds.19All the participants were current residents of Penamacor. The group included, among others, former workers, a journalist, people connected to local politics, and people involved in cultural organizations. We presented materials collected during archival research, encouraging an open debate. We asked about selected buildings – namely the Casa do Povo, the market hall, and a secondary school – and the students shared their memories about their use and transformation. Attendees were successfully engaged in this activity, and this allowed us to establish contacts for further in-depth interviews. We were also led to better define the social, cultural, and economic importance of these facilities.

**Figure 2:**
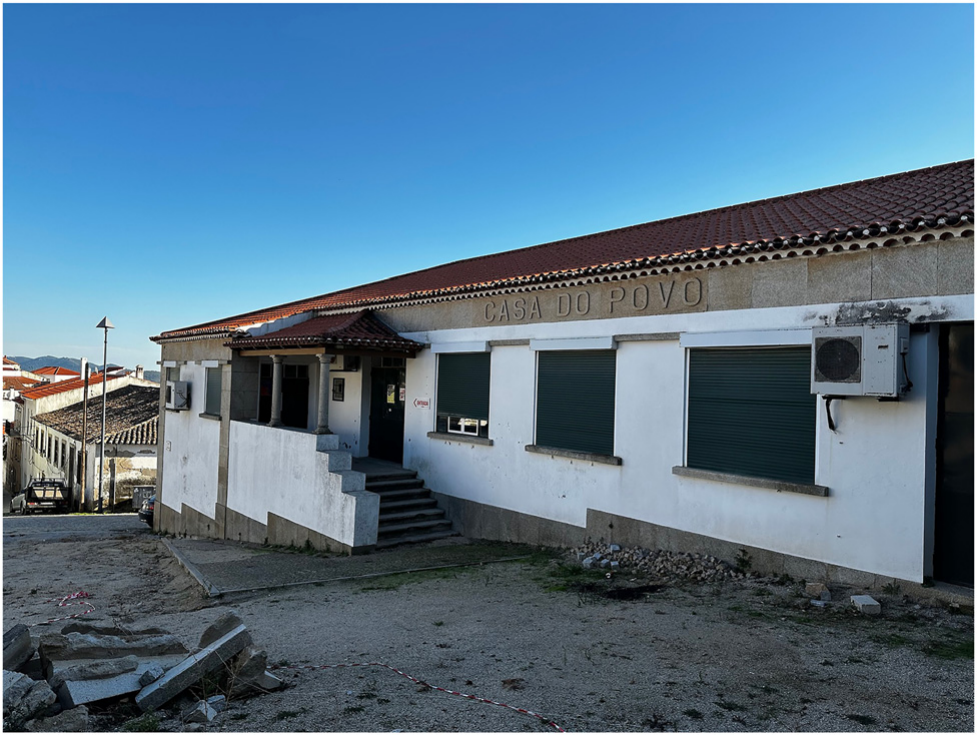
The Casa do Povo building in Penamacor, 2024. Copyright: @ArquitecturaAqui.

During that event, the Casa do Povo building (Figure 2) was singled out for its historical and social heritage relevance. However, it was only on a second fieldwork trip that the significance of the Casa do Povo for Penamacor’s everyday life became clear. This facility, finished in 1953, is still in use today for various activities, but its history goes back several decades. The establishment of a Casa do Povo in each rural community in Portugal had been a central tenet in enacting social control and assistance policies during the Estado Novo dictatorship regime (1933–1974): a local mutuality supported by the mandatory contributions of farmers provided members with medical and social welfare aid, and cultural, educational, and recreational activities, while also allowing for a degree of socio-political control to be exerted by the central State.20See e.g. Dulce Freire, “Estado Corporativo em Acção: sociedade rural e construção da rede de Casas do Povo,” in *Corporativismo, Fascismos, Estado Novo*, ed. Fernando Rosas and Álvaro Garrido (Coimbra: Almedina, 2012), 273–302; Manuel Lucena, “Casas do Povo,” in *Dicionário de História de Portugal*, vol. VII, eds. António Barreto and Maria Filomena Mónica (Lisboa: Livraria Figueirinhas, 1999), 245–50; Diego Inglês de Souza et al., “O Presente e o Passado das Casas do Povo em Portugal: Arquitetura, Comunidade e Memória,” *Revista ARA* 15, no. 15 (2023): 73–107. Unlike other regime institutions that were abolished when democracy was reinstated in Portugal, the Casas do Povo were appropriated by communities and turned into non-governmental associative bodies, generally focused on cultural and recreational ends.21In some cases, the organizations were abolished, and the buildings were appropriated by the Social Security Institute. During our second stay, Francisco proposed a visit to the Casa do Povo building, where he and the president of the Junta de Freguesia – who is also a Penamacor long-term resident – explored the transformations and uses of this building over time, as well as its present uses and organization, uncovering a deep emotional connection to the site during the semi-structured interviews. Furthermore, a group conversation organized by Francisco Abreu with residents and Casa do Povo users provided a deeper insight into its significance to the community, while a collective walk through the town offered a broader perspective on the social context of this facility. The group was composed of two educators, male and female, in their late middle age, and a group of eight elderly inhabitants with different backgrounds.

These conversations evidenced that the Casa do Povo was not merely an institutional place but, above all, a meeting place that has energized the social fabric in Penamacor over the years. Archival research limited our knowledge to construction plans and dates. In contrast, the conversations exposed the central role of the medical care provided by the organism, given the absence of other healthcare providers nearby; our hosts revealed the location of the former doctor’s office. Furthermore, people emphasized the social relevance of the organism and the role of its president particularly after the 1974 democratic revolution, when farmers were encouraged to join the Casa do Povo in order to receive a retirement pension. In the aftermath of the revolution, the building became the stage for political rallies. Participants also shared the cultural and socializing uses of the building throughout time: we learned about the extension of the facility in the 1970s to increase the capacity for hosting celebrations such as weddings and parties; since then, the premises have been used as a gym for students, hosted a music school that catered to the youth of Penamacor, as well as informal clubs and the folk dances group, which is still functioning. The value of this building remains above all in its cultural and popular purposes: it is strongly associated with everyday experiences, where multiple collective activities take place.

**Figure 3:**
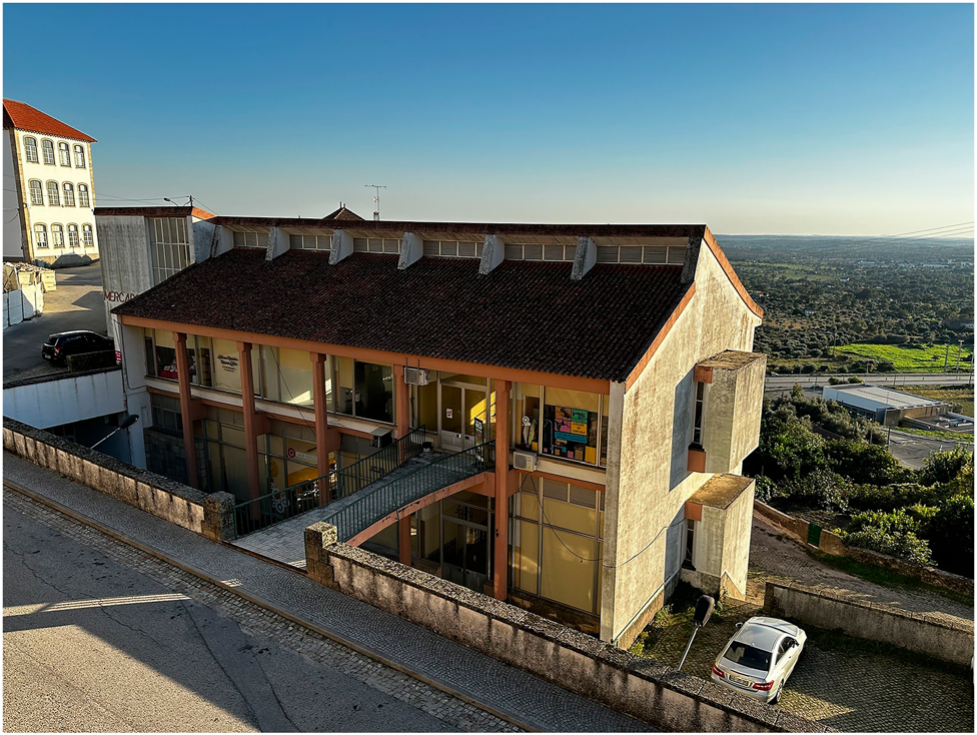
The municipal market hall in Penamacor, 2023. Copyright: @ArquitecturaAqui.

The lively use of the Casa do Povo building today contrasts with the obsolescence and disrepair of the municipal market hall, which was also highlighted for its historical complexity during the event at the senior academy. The market hall (Figure 3) is another emblematic building in Penamacor, centrally located near its public gardens. Archival documentation depicted the history of its commission and construction process: the market hall was planned since the late 1960s by an initiative of the municipality and was completed in 1979. As a resident recounted, the delay in the construction was attributed to several factors, including a disagreement between the municipality and the designer, which was not documented in the sources. Although still a landmark of modern architecture in Penamacor, the market hall has been gradually losing its relevance for the community, facing obsolescence and the loss of social and economic prospects. We were able to combine the memories shared by former users at the senior academy with the current experiences of two middle-aged female retailers who remain on site. Once a central structure in the local economy as a place for farmers to sell their produce, the market hall is no longer relevant for trading essential goods, mainly since supermarkets were introduced following stricter regulations for commercializing fresh produce. Depopulation was also blamed for the decline. Nowadays only the upper floor is being used by small shops, as structural problems affect the ground level: according to the senior academy participants, humidity problems occur as the market hall was built on the site of a cistern. Thermal comfort is another issue, given the limited performance of the structure when facing substantial variations in outdoors temperature between summer and winter (for example, one shopkeeper noted that it is not possible to open the large windows, so she had to install air conditioning). The butcher is the only shop still related to the original function of the building and mainly serves elderly customers. Surviving commercial areas include a seamstress, a hairdresser, and a haberdashery; other tenants, such as a real estate agency, a restaurant, and a charity shop, have closed their businesses. In 2024, the future of the market hall remained uncertain. The information provided by the users allowed us to co-create a new narrative, widely accessible for all to read and improve upon, that sheds light on socially relevant aspects such as its relation to the commercial fabric, the local demographic situation, and the evolution of the building and its functions over the years.

Our experience in Cáceres (https://arquitecturaaqui.eu/es/comunidades/2286/caceres), in turn, carried other challenges: this is a large city, densely populated, where access to and engagement with community members were harder to secure than in Penamacor.22In 2024, the city of Cáceres had 96,441 inhabitants, and the municipality encompassed 387,820 inhabitants. Data obtained at Instituto Nacional de Estadística (INE), Spain. Cáceres is a provincial capital in the autonomous region of Extremadura, Spain; today, a tourism destination known for its “monumental” heritage, with the economy relying mostly on the service industry.23Cáceres was listed as UNESCO World Heritage in 1986 for its cohesive urban nucleus from the Middle Ages and Renaissance. Agriculture was the economic base throughout most of the twentieth century, stimulating migration to the periphery of more industrialized Spanish cities, as well as to Europe, between the 1950s and mid-1970s; the phenomenon of return became a reality in the 1980s.24Gonzalo Barrientos Alfageme, “Las Migraciones Extremeñas en el Último Cuarto del Siglo XX,” *Revista de Estudios Extremeños* 63, no. 3 (2007): 1311–30. In terms of collective-use facilities, the development of social housing and schools was predominant between the 1950s and 1980s, with occasional construction of administrative buildings and welfare facilities. After the initial stage of data collecting and archival research, two major themes regarding state intervention were identified: the prominence of educational facilities relating to social needs, and the vast amount of subsidized housing units built. The first type of buildings deals with questions of education, deprivation, and social control, both during the Franco dictatorship and during the democratization process. The second relates to a basic societal requirement that needed addressing to improve living conditions and accommodate populational growth, particularly from the 1960s onwards.

Fieldwork, including local archival research, was conducted between July 2023 and April 2024.25In Cáceres, archival research was conducted at two municipal archives, as well as the historical and administrative archives of the provincial government of Cáceres. Given the size of the municipality, the participation strategy focused on interviews with different actors, rather than on group events. First, we held conversations with both a local archivist – who is also the chronicler of the city – and an architect from the municipality who wrote a doctoral thesis on Cáceres’ urban development, which provided us with an insight into the general context of the city. Fernando Jiménez Berrocal, the archivist, shared his publications about the history of some of the city’s buildings. Carlos Sánchez Franco, the municipal architect, contributed to revising our fieldnotes, providing his input as a researcher on the subject. Furthermore, Carlos revealed interest in our platform and is considering incorporating data from our research into the municipal cartography database.26Spatial Data Infrastructure of Cáceres (IDE). See https://ide.caceres.es/.

Afterwards, we concentrated on specific buildings, targeting relevant users for interaction and analyzing the cases separately. Among others, the former Universidad Laboral building complex and the “Las Trescientas” housing unit were analyzed: for these cases, four semi-structured interviews and three informal conversations were conducted.

**Figure 4:**
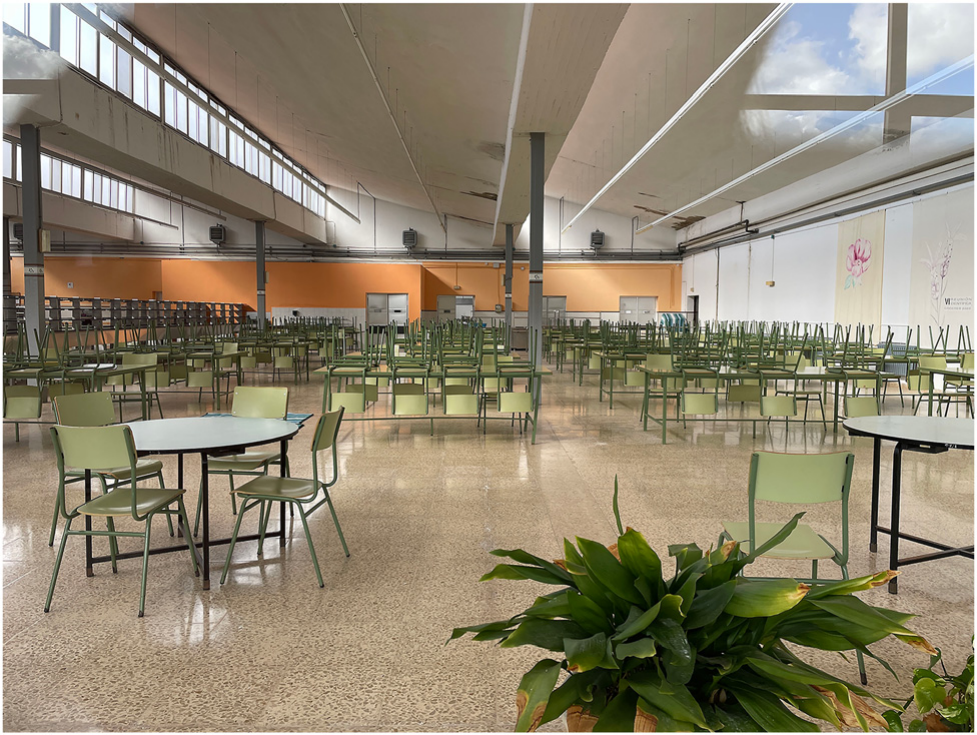
The Universidad Laboral secondary school in Cáceres, 2024. Copyright: @ArquitecturaAqui.

The now-called Universidad Laboral secondary school is part of a complex of buildings erected in the 1960s to accommodate the city’s workers university (Figure 4). Worker universities were created by the Francoist regime to provide specialized education to the working classes, while simultaneously attempting to ideologically indoctrinate them.27Patricia Delgado-Granados and Gonzalo Ramírez-Macías, “¿Conveniencia o necesidad? La formación de la clase obrera en las Universidades Laborales franquistas (1955–1978),” *Historia Crítica* 1, no. 63 (2016): 117–36. The initial intent of the worker university in Cáceres was to specialize in agricultural and forestry training, which was never achieved. Two in-depth interviews were possible in a second fieldtrip, having been facilitated by the director of the school, who was contacted via e-mail after initial attempts to contact her on site. After presenting our project in an informal telephone conversation, we were referred to two former students, a middle-aged woman and an older man, who are now teachers at the school. Once again, the gatekeeper’s selection produced interesting backgrounds to understand the singularities and the evolution of the facilities.

The director’s intermediation role proved essential in facilitating visits to the building, which can involve navigating bureaucratic intricacies. The interviews were made on site, enabling the direct recollection of spatial memories, evoking aspects of everyday life and experiences across generations that are not patent in official archival documents, while observing current uses of the facilities. The shared information goes beyond the architectural value of the buildings, whose rationalist features and designers have been highlighted.28Docomomo Ibérico: https://docomomoiberico.com/pt-pt/edificios/universidad-laboral-hispano-americana/(accessed 17.01.2025); Pablo Basterra Ederra, “Las Universidades Laborales de Luis Laorga y José López Zanón: Estudios sobre el Módulo,” *Ra. Revista de Arquitectura* 18 (2016): 89–96. The interviewed revealed important details about the institution: the progressiveness of teaching during the 1970s, at a moment when this was one of only two worker universities for female students in Spain; having been conceived to receive students from other provinces, not all were boarding students; university-level courses were taught during that period, namely a school for business studies and a nursing school, which were not exclusive for female students. In 1977, it became a mixed school.

Memories of daily life at Universidad Laboral were also conveyed: movie screenings on weekends in the main hall, lively mealtimes at the large cafeteria with a sophisticated dish collection system, the bus that brought external students to school (the city being quite distant). Interviewees described current uses of the facilities and interventions (such as closing a patio); the fact that Cáceres’s only Olympic-size swimming pool, located on the premises of the school, is closed due to lack of funding for maintenance was particularly noted. Our research and conversations went beyond the common narrative, highlighting the modernity of the architecture and its authors and bringing to light the socio-political importance of this facility. The modernity of this facility was not only architectural, it turns out: it was reflected in novelties for the time, such as an astronomical observatory and well-equipped chemistry laboratories. In the social life of students, this idea of modernity was reproduced and translated into the possibility of critical thinking and academic excellence, which implied effort and strict discipline. These aspects of this ensemble had not been unveiled before.

**Figure 5:**
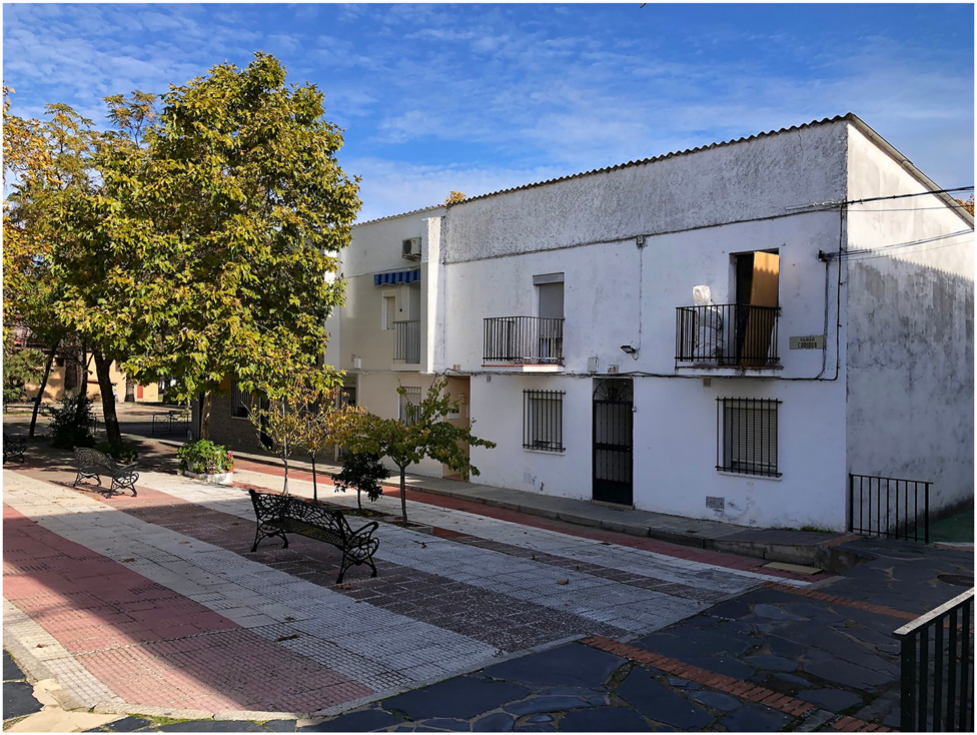
“Las Trescientas” housing scheme in Cáceres, 2023. Copyright: @ArquitecturaAqui.

We conclude this account of our Cáceres research with the housing estate known as “Las Trescientas” (Figure 5). The scheme was built in the 1960s in the then outskirts of Cáceres as an *Unidad Vecinal de Absorción*, an emergency-response scheme to eliminate shanty towns, as well as to accommodate people whose properties had been expropriated. The state attempted to tackle the shortage of housing for low-income classes by promoting construction. “Las Trescientas” refers to the 300 single-family homes planned for this neighborhood, a peculiar choice of low-rise features and terraced housing solution with village-like streets and squares, in contrast with the denser fabric of social housing blocks that surrounds it. Planned as a temporary accommodation but built with concrete elements and other perennial materials, the scheme is still in full use today.

In our second fieldwork mission in Cáceres we contacted Jorge Civantos, son of the architect Tomás Civantos, who designed this neighborhood. Jorge is a well-known *arquitecto técnico*29An academic degree used in Spain which roughly corresponds with the degree of architectural technologist in the United Kingdom. interested in preserving Cáceres’ local heritage. We contacted him through the regional architects’ union site, and he was very collaborative from the start. He invited us to his home and shared documentation from his father’s estate on this scheme’s construction process, providing us with digital copies, a valuable resource, as this documentation was not available in any public archive.

This experience reveals that the characteristics of our contacts are fundamental. Jorge has an impressive trajectory as an architect, but only had indirect knowledge of the construction process of this scheme through his father’s recollections. Nevertheless, he made a generous effort to remember what his father valued and frequently repeated about architecture and this neighborhood, so that we could understand the architectural value of this complex. He shared information on the design, construction process, expected uses, and needs that the architect aimed to address. At one point, he showed us a film of the architect during the construction, which expressed the intersection between biography and architecture. This scheme was one of his father’s first projects. Tomás Civantos was young and enthusiastic and brought to the design many of the interests he maintained throughout his career. Reading this project with his son, we discovered issues such as his interest in thermal control, which he tried to solve with limited budgets. We also discovered his interest in user experience, in elements such as the design of the streets and squares surrounding the houses, aimed at promoting gatherings and proximity among neighbors, and the kitchen windows that allow a short person or a child to look out onto the street. Tomás Civantos gave the workers freedom in the design of the breeze blocks to express their creativity.

When asked about the experience of living in “Las Trescientas,” Jorge was clear: we should speak to residents, who have a direct experience. This showed both humbleness and a less common belief among architects: that history of architecture goes beyond the figure of the architect or written documents. Accordingly, he extended his invaluable support by contacting a family living in this neighborhood for an interview. The next day, he introduced us and joined us at the beginning of the conversation, helping to establish trust (*rapport*, in anthropology). This interview shed light on details about daily life, personal processes, and collective experiences lived in the neighborhood that illustrate its evolution.

Even though the scheme was intended for lower income classes, mainly to rehouse families who had previously lived in shantytowns, socio-economic diversity was a reality. The woman in the family we interviewed had no previous experience of living in low-rise, village-like houses, as she came from a neighborhood with blocks of flats on the outskirts of the city, La Pinilla. The family noted that the previously common socialization among residents has declined as people grew older and new generations occupy the houses. The closure of neighborhood shops also contributed to this change. Nonetheless, the local neighbors association is still active. The woman stressed that her situation of caring for family members led to a markedly different experience from other residents, who usually highlight the daily encounters that took place in courtyards or squares. Her daily life, by contrast, was spent mostly indoors and not in public spaces that were well adapted for daily interaction. “Las Trescientas” is nowadays completely integrated with the urban fabric, close to public services and not isolated in the outskirts as in its origin. The renting scheme established in the beginning was also subverted, as residents had the opportunity to acquire the houses from the municipality in the 1990s. As in previous experiences, the history of this neighborhood was understood in a comprehensive manner by involving people with different backgrounds who can elucidate aspects of everyday life. Therefore, the scheme appears as an object in constant evolution, from the construction project itself, through its implementation, to its daily use over several generations.

In both communities, devolution consisted in sharing conversation notes for review and maintaining dialogue, updating people on the latest developments on the platform. Some participants shared material with us and plan to use our data for public dissemination. All contributions are reflected on our platform *Arquitectura Aqui*, which has been shared to encourage further input; while most participants showed interest, tangible contributions are still forthcoming.

## 4 Discussion: Towards a Collective Knowledge Construction

Participation and engagement with local communities are part of a multi-stage process, starting with initial contacts with the communities, followed by a relationship of mutual familiarity, and a final discovery and communication of shared knowledge. Our strategy is based on established methodologies in architectural history and ethnography, adapted to contingencies of historical research and fieldwork, and open to a degree of experimentation. Different types of conversations give us a glimpse about the role of the past in the community and allow us to collect memories. Our research pursues a balance between facts retrieved in archives and present-day personal recollections. To do so, we include lesser-acknowledged voices and experiences of individuals and groups within these communities as a way of rewriting history. Through conversations with neighbors, we aim to give voice to multiple perspectives capable of highlighting the cultural, economic, and socio-political significance of buildings that have historically remained little explored. This is an attempt to build a situated knowledge to redirect the construction of history to new agents and social concerns, giving visibility to experiences that people value.

However, this work has entailed several challenges that we creatively resolved, mainly the time constraint for conducting fieldwork on-site. Since anthropology understands fieldwork as a deep immersion in the everyday practices, a more limited time frame represented a great challenge. The information gathering took a prominent role, and the whole research process relied mostly on the researcher. In contrast to assembly processes or long-term collective participation, our fieldwork could not establish a substantial horizontal relationship with the citizens. For example, it was the researchers who often articulated meaning through people’s contributions. Nevertheless, it is the compilation of diverse and equally valid voices that allows meaning to be narrated and interpretated. Furthermore, the opportunity for contact comes often not from one or two, but from several people who contribute information according to their possibilities. Therefore, despite time limitations, citizen collaboration allowed us to gather various voices.

Limited time often results in initial suspicion from some community members and hinders the gaining of trust. Thus, our research highlights the importance of the available social links to the community. We address this by first identifying potential collaborators, while also trusting the potential partners that we meet on the ground, highlighting people with close and non-formal links to the community. This article evidenced that the gatekeeper is frequently a crucial actor. The examples aim to highlight the capacity of each person to build knowledge from their specific circumstances and at different levels. The trust placed in our gatekeepers has allowed our work to be better understood and valued from the outset, encouraging more people to share their knowledge.

The strategy adopted for local engagement in the two communities also benefitted from previous fieldwork experiences. In our practice, organizing events with specialists, experts, and local authorities – which we have done in the context of our research – tends to prevent citizens from interacting as they believe that they have nothing to add or that they know nothing about the buildings, which limits participation and co-creation of knowledge. By contrast, the proximity established with certain actors led to much richer and more varied experiences. In Penamacor, Francisco – who is a neighbor and not only a formal agent – acted as a facilitator, enabling us to walk through the village and learn *in situ* from community members and their interests. This is quite different from organizing top-down guided tours to visit buildings where the audience tends to passively receive information.

Another strategy has been to focus on specific groups within the community since it proves to facilitate collaboration: for instance, the residents of a scheme, or vendors at a market hall. Likewise, the study of specific buildings often facilitates memory retrieval and even provokes surprise and curiosity, as they are perceived as historically forgotten objects. This astonishment at our presence as researchers often translates into hospitality as people involved in their community find our work valuable for collective memory. As Jesse Adams Stein remarked when writing on the co-construction of a “collective spatial memory” in interviews with building users, the process is a site where meaning is made, involving interaction between interviewer and interviewee.30Stein, “The Co-construction of Spatial Memory”: 180. Our research confirms that memories of working, studying, or living in a specific building are deeply connected to the spaces where those routines took place. They are particularly evoked when the conversation occurs in or near the building. These experiences promote a dialogue between science and citizenship as a collective and collaborative process of knowledge construction, discovering unknown stories and even mutually rewarding partnerships.

Certain constraints are inevitable, particularly due to the limited time available to interact with each community during fieldwork. We have noted a persisting social distance between some community members and us, both in rural communities and in cities, as a result of time constraints and the several steps usually involved in trust building. In the discussed cases, people were more available to engage in a spontaneous conversation in the smaller community of Penamacor, while in Cáceres, we were generally asked to first address the director or the person responsible for the building we visited. As noted, contacts are often established at a distance and in great advance, which can be a hindrance to effective engagement. After these first, generally remote contacts, personal presence and face-to-face conversations proved essential for further collaboration.

Moreover, we must be aware of another challenge we face as researchers, namely the distance – both geographical and institutional – separating the university from the communities being studied, which can create an overlap between worlds. Collaboration with communities supported connection between academia and citizens, enabling engagement with meaningful and critical issues relevant to the latter. Ideally, we return several times to each community in a process of devolution, seeking validation of the information digested and presented online, and for a final presentation of the work accomplished. As Gardner and Hamilton noted, using the internet as the media to disseminate public history does not always entail a more democratic access.31James B. Gardner and Paula Hamilton, “Introduction. The Past and Future of Public History: Development and Challenges,” *The Oxford Handbook of Public History*, 1–22, 13. We consider that the final, in-person presentation is essential for disseminating public history to older, less digitally literate individuals who may not have had access to our platform previously. However, this process is not always feasible due to our intense work schedule. Aware of this limitation, we recontact some interviewees to update them on the project via phone or email, or indirectly through key informants who do visit the platform. These contacts usually lead to further conversations and revisions of recorded notes or writing drafts, drawing on, and nurturing, personal bonds. While this strategy may be less than ideal, it still provides some reciprocity towards the people who have given us their support. The established bond of trust is fragile and needs to be carefully handled as it may be strengthened or weakened according to our level of engagement in the community.

In public history-making processes, it is essential that the concerns of the people we meet find a space to be communicated with mutual appreciation and respect. In Penamacor, for example, our encounter propelled some of Francisco’s own interests and allowed for a real connection to be established between research and citizenship: a natural link between his longtime interests and our pursuit of citizen history. In our approach, people are often encouraged to join the process of creating new and more diverse representations of history. Francisco had already made efforts to preserve the collective memory of the town by writing several books on local history, and having a sharp ethnographic eye for everyday activities and places. He even invited us to a familiar and well-known place in the town, a now closed tavern that had been run by his parents. All this information can be overlooked by a researcher who is not attentive to the day-to-day experiences, hence the importance of actively listening and recognizing the value of the contributions. Our ethnographic approach allows us to consider symbolic constructions as an important part of history and prevents us from reproducing a new distance that would not validate this type of knowledge. The ethnographic perspective helps to bridge the gap between everyday life and academic language, connecting holistically with less acknowledged voices and under-studied objects. At first sight, it is scattered and heterogeneous information, but it finds a place in our research when the voices of those constructing the collective memory are legitimized as valid narratives. Local people highlight matters of public interest that we were not aware of, and that we would be unlikely to find out without their collaboration.

We held less conversations in Cáceres than in Penamacor, but both cases revealed a rich diversity of microhistories beyond the archives. We suggest that this is possible given our receptiveness towards the skills and resources that local people were willing to provide. The process of collective knowledge construction is informed by a multitude of personal circumstances manifesting in diverse forms, ranging from subtle clues in casual conversations to the welcome offered by a host. We are committed to translating disparate experiences and narratives into a public contribution, as a response to people’s constant efforts to preserve their local history. Francisco frequently expressed his surprise and satisfaction at our decision as outsiders to study Penamacor. These experiences are crucial, particularly for recognizing and legitimizing the value of shared knowledge. This, we posit, is research as a grassroots construction.

Our exercise opened relevant prospects on the communities’ social history by tapping into the relationships between everyday life and architecture and approaching the historical development from the standpoint of residents. Concurrently, the impact of recent societal changes became evident, such as those in Cáceres: access to education and housing was shown to be a complex process that helped the lower classes to improve their situation and strengthen their role in building the city. Most of the students of the Universidad Laboral have gone on to work in the service sector, particularly in administration, banking and the civil service, which is consistent with the city’s development in the late twentieth century. Conversely, many of the residents of “Las Trescientas” were former peasants, slum-dwellers, or inhabitants of the expanding urban fringes of Cáceres. The scheme is now fully integrated into the urban fabric of the city, but its design evokes the architecture of a recent past, specifically its connection with the countryside. The Universidad Laboral was designed for agricultural studies and now is surrounded by open fields, a reminder of its link with the rural world. In fact, Cáceres itself is still surrounded by countryside, revealing diverse social and spatial configurations and ambivalences.

In Penamacor, in turn, we observed two facilities at different levels of use to acknowledge their historic and current relevance. Penamacor is a remote town with poor transportation connections that has been severely impacted by the exodus of young people. However, we gained insight into how daily efforts are made by residents to maintain social, cultural, and economic life. The Casa do Povo’s varied activities promote an active social life in the community. These types of buildings are particularly interesting for understanding how community dynamics allow for a more authentic democratic transition process at a local level. The Casa do Povo has overcome the institutional boundaries associated with its establishment under the dictatorship, evolving into an everyday meeting place following the changes experienced by Portuguese society over the last five decades. Although both the Casa do Povo and the Universidad Laboral were created to implicitly exert social control, this might not have been perceived as such by all users at the time and has clearly been lost since. The market hall also reflects the efforts of traders and customers who invest in this type of commerce, giving it a presence and enabling its maintenance. It also reveals striking paradoxes: some of the services communities require today, such as supermarkets, threaten an entire socio-economic structure of proximity that is key in places such as these.

We hope that this process will be of mutual benefit to both parties. To this end, we make our skills as scholars available to the public. The platform *Arquitectura Aqui* is being built as a meeting place for both scholars and the public. It includes records of the conversations, which, together with data drawn from archives and documentation, is integrated into the description of buildings and communities. In this way, we attempt to bridge the gap between the written and the oral, the official and the informal. We do not yet know the real impact of this platform on built environment management strategies, but we do know that many people appreciate it and the visibility given to their everyday surroundings. We encourage community members to participate with improvements, comments and suggestions, thus nurturing the platform over time.

## 5 Final Remarks

These buildings, public by definition and constructed through public funding, gain meaning through lived experiences that can be openly shared as public knowledge. Understanding communities from below and considering their relation to collective-use facilities over time, regarding experience as an essential part of a broader narrative, seems fundamental in going forward, towards a public history of architecture.

The presented cases reveal interesting patterns for constructing a story. First, trust building must be based on mutual interest, as the research is not only more ethical but also more fruitful, because it enables a shared creation of knowledge by bringing together people who make different contributions. Secondly, we illustrate the need to open architectural history beyond the drawn and written sources of production to encompass the records of use. A public history proposal based on an inclusive approach allows citizens to overcome initial suspicions, generating interest and mutual appreciation. People guide us towards new themes and research lines, shaping the general – and somewhat abstract – researchers’ concepts into something embedded in local history. Community members are crucial for co-constructing a public history: they provide access to researchers, contextualize problems, and allow us to construct research objects, discovering previously unknown or under-recognized histories.

Through this back-and-forth process of dialogue, we intend to reveal that these facilities are complex historical objects. Beyond the physical building and documentation, everyday life is expressed through memories, (un)known events, and personal and collective experiences that connect biography, territory, history, and architecture. We aim to build a public history that contributes to the idea that architecture is socially relevant, highlighting the value that communities give to public facilities. We want to follow in people’s footsteps, tapping into their skills, efforts, and hopes to shape the histories of these places.

